# Effects of Novel Multimodal Transversus Abdominis Plane Block on Postoperative Opioid Usage and Hospital Length of Stay Following Elective Ventral Hernia Repair

**DOI:** 10.7759/cureus.38603

**Published:** 2023-05-05

**Authors:** Alexandra C Skoczek, Patrick W Ruane, Alex P Rasarmos, Dennis L Fernandez

**Affiliations:** 1 Medicine, Edward Via College of Osteopathic Medicine - Auburn, Auburn, USA; 2 Medicine, Edward Via College of Osteopathic Medicine, Spartanburg, USA; 3 General Surgery, Crestwood Medical Center, Huntsville, USA

**Keywords:** in-hospital length of stay, visceral pain, pain management, neuromuscular blockade, ventral hernia, nerve block, opioid analgesics, acute postoperative pain

## Abstract

Background and objective

Traditional transversus abdominis plane (TAP) blocks consisting of a local anesthetic, typically bupivacaine, have previously been shown to reduce postoperative pain following gastrointestinal surgery, including hernia repair. However, elective abdominal wall reconstructions for the repair of large ventral hernias continue to cause patients significant postoperative pain, resulting in prolonged hospital stays and need for opioid pain medication. This study aimed to analyze the postoperative opioid pain medication usage and hospital length of stay (LOS) in patients who received a nontraditional multimodal TAP block of ropivacaine (local anesthetic), ketorolac (non-steroidal anti-inflammatory), and epinephrine following elective ventral hernia repair.

Methods

A retrospective review of medical records for patients who underwent elective robotic ventral hernia repair by a single surgeon was conducted. Postoperative hospital LOS and opioid usage for patients with the multimodal TAP block were compared to those without.

Results

A total of 334 patients met the inclusion criteria for LOS analysis: 235 received the TAP block and 109 did not. Patients who received the TAP block had a statistically significant shorter LOS compared to patients who had no TAP block (1.09 ± 1.22 days vs. 2.53 ± 1.57 days; P<0.001). Medical records for 281 patients, 214 with the TAP block and 67 without the TAP block, contained information and were analyzed for postoperative opioid usage. A statistically significantly fewer number of patients who had the TAP block required hydromorphone patient-controlled analgesia pump (3.3% vs. 36%; P<0.001) and oral opioids (29% vs. 78%; P<0.001) postoperatively. Those with TAP block required intravenous opioids more frequently (50% vs 10%; P<0.001) although at much less dosages than those without TAP block (4.86 ± 2.62 mg vs. 10.29 ±3.90 mg; P<0.001).

Conclusion

In conclusion, this multimodal TAP block of ropivacaine, ketorolac, and epinephrine may represents an effective method to improve hospital LOS and postoperative opioid usage in patients undergoing robotic abdominal wall reconstruction for ventral hernia repair.

## Introduction

Transversus abdominis plane (TAP) blocks have been shown to effectively reduce postoperative pain following gastrointestinal surgery, including hernia repair [1,2]. Postoperative pain following hernia repair is influenced by the size of the abdominal wall wound and the extent of intra-abdominal dissection [1]. With the advancements of abdominal wall reconstructions (AWRs), patients have begun to undergo elective repair for larger more advanced ventral hernias requiring more extensive abdominal dissection and leading to increased postoperative pain [3]. Postoperative pain continues to negatively affect postoperative opioid requirements and hospital length of stay (LOS), making pain control a surgical challenge of high importance [4,5].

TAP blocks are performed by injecting a long-acting local anesthetic agent, typically bupivacaine, in the neurovascular plane between the layers of the internal oblique and transversus abdominis muscles targeting the intercostal spinal nerves [1,2,6-8]. The local anesthetic spreads within this plane to block the neural afferent signaling of the thoracolumbar nerves, providing therapeutic analgesia to the anterolateral abdominal wall [9]. TAP blocks can be placed either under ultrasound guidance or intraoperatively with direct visualization under laparoscopic guidance. While these methods can confirm the correct placement of the block, the total area receiving analgesia cannot be well controlled and is influenced by anatomic variation, injection volume, and choice of approach [9].

Local anesthetics combined with epinephrine and a non-steroidal anti-inflammatory drug (NSAID) when used in large volume as local analgesia for orthopedic procedures have been shown to reduce postoperative pain scores and the use of opiate medications [10-12]. The addition of epinephrine and an NSAID may contribute to additional analgesia due to extended analgesia duration, delayed systemic uptake, and decreased postoperative gastrointestinal complications [13-17].

Currently, no studies have been conducted analyzing the effects of a multimodal TAP block on minimally invasive gastrointestinal surgery. This study aimed to analyze the postoperative opioid pain medication usage and hospital LOS in patients who received a nontraditional multimodal TAP block of ropivacaine (local anesthetic), ketorolac (non-steroidal anti-inflammatory), and epinephrine following elective AWR for large ventral hernia repair. It was hypothesized that the incorporation of this nontraditional combination TAP block would lead to a decrease in opioid usage and a shorter hospital LOS postoperatively.

## Materials and methods

Data collection and study design

After obtaining an exemption from the institutional review board (Edward Via College of Osteopathic Medicine, IRB #2022-079), patients who had undergone an elective robotic AWR for primary ventral hernia repair between January 2015 and May 2022 were queried. A single surgeon performed all cases using the same surgical technique. Hernia repair was done using the robotic transversus abdominis release method with resorbable biosynthetic mesh underlay. Patients whose procedures were converted to a laparoscopic or open procedure and patients with incomplete medical records were excluded. Demographic information, perioperative procedural data, and postoperative data were collected from a prospectively managed electronic health record system.

TAP block technique

A multimodal TAP block containing 246.25 mg (49.25 mL) of ropivacaine, 30 mg of ketorolac, 0.5 mg of epinephrine, and 0.9% NaCl quantum satis to 200 mL was prepared by the pharmacy prior to the onset of surgery. The dosage of ropivacaine, ketorolac, and epinephrine are consistent with the dosages used in previously studied orthopedic procedures [10,11]. Normal saline was used to increase the total volume to 200 mL in order to completely saturate the abdominal wall. With the patient in the supine position, an incision was made and a robotic camera port was placed. A camera was then inserted to visualize the abdominal wall during the administration of the TAP block. Under direct visualization, the needle was advanced through the skin at the midclavicular line and then further advanced into the TAP. Correct placement of the TAP block was confirmed by visualization of a bulge after injection. The procedure was then repeated ipsilaterally at multiple positions in the midclavicular line between the iliac crest and the costal margin. The block was then performed on the opposite side using the same technique. The 200-mL TAP block was equally split between the two sides. Previous clinical trials of TAP blocks using just a local anesthetic have varied in injection volumes between 50 and 100 mL on each side dependent on incision size [9,11].

Outcomes of interest

The main outcomes of interest were LOS, prolonged LOS, postoperative hydromorphone patient-controlled analgesia (PCA) pump usage, intravenous (IV) opioid usage, and oral opioid usage. Prolonged LOS was defined as >4 days as the average reported LOS following hernia repair in the literature is 2-4 days [18]. Data were reported as numbers and percentages for categorical variables and means, standard deviation, medians, and interquartile ranges for continuous variables where appropriate. Univariate analysis comparing the two groups (those who received the TAP block and those who did not) was performed using the Pearson chi-squared test for categorical variables, the Fisher exact test for zero counts, and the Kruskal-Wallis test or analysis of variance for continuous variables. Statistical analysis was conducted using R: The Project for Statistical Computing [19].

## Results

Study participants

A total of 344 patients met the inclusion criteria for LOS analysis. Of those, 68% (n = 235) had received the multimodal TAP block and 32% (n = 109) had not received the TAP block. The change in TAP block usage was due to a change in surgical technique and surgeon preference over time. Univariate comparisons of patient demographics including age, advanced age (>70 years old), and gender, as well as abdominal surgery history, hernia characteristics, and body mass index (BMI) are presented in Table 1. The mean BMI for the population was 34 ± 7 kg/m^2^ with no significant difference seen between the TAP block and no TAP block groups (34 ± 7 kg/m^2^ vs. 35 ± 9 kg/m^2^, P = 0.69). Mean hernia diameter for the population was 12.7 ± 5.2 cm with a mean area of 147 ± 133 cm^2^. No significant difference was seen between the two groups for hernia diameter (12.5 ± 4.8 cm in the TAP block group compared with 13 ± 5.8 cm in the non-TAP block; P = 0.20) and hernia area (142 ± 114 cm^2^ in those with the TAP block compared with 159 ± 165 cm^2^ in the group with no TAP block; P = 0.49).

**Table 1:** Demographics - length-of-stay analysis ʄKruskal-Wallis test; ɸPearson's chi-squared; ǂAnalysis of variance test TAP, transversus abdominis plane

Characteristic	N	TAP Block, N = 235	No TAP Block, N = 109	Combined, N = 344	Test Statistic
Age (median [Q1-Q3])	344	57 (47–67)	58 (45–67)	58 (46–67)	P > 0.9^ʄ^
Advanced age (>70 years)	344	17% (40)	17% (18)	17% (58)	P > 0.9^ɸ^
Gender	344				P = 0.11^ɸ^
Female		64% (150)	72% (79)	67% (229)	
Male		36% (85)	28% (30)	33% (115)	
Number of previous abdominal surgeries (mean ± SD)	344	2.51 ± 2.08	2.51 ± 1.85	2.51 ± 2.01	P = 0.70^ɸ^
Hernia diameter in cm (mean ± SD)	344	12.5 ± 4.8	13 ± 5.8	12.7 ± 5.2	P = 0.20^ǂ^
Hernia area in cm (mean ± SD)	344	142 ± 114	159 ± 165	147 ± 133	P = 0.49^ǂ^
BMI (mean ± SD)	344	34 ± 7	35 ± 9	34 ± 7	P = 0.69^ǂ^

Of the 344 patients included in the LOS analysis, data on postoperative opioid usage were able to be obtained from 281 of the patient’s charts. Of the 281 patients included in the opioid analysis, 75% (n = 214) received the multimodal TAP block and 25% (n = 67) did not receive a TAP block. Univariate comparisons of patient demographics including age, advanced age (>70 years old), and gender, as well as abdominal surgery history, hernia characteristics, and BMI are presented in Table 2. The mean BMI for the subpopulation was 34 ± 7 kg/m^2^ with no significant difference seen between the TAP block and no TAP block groups (34 ± 7 kg/m^2^ vs. 34 ± 8 kg/m², P = 0.50). Mean hernia diameter for the subpopulation was 12.5 ± 5.0 cm, with a mean hernia diameter of 12.5 ± 5.0 cm in the TAP block group and 12.5 ± 5.2 in the non-TAP block group (P = 0.42). The mean hernia area in the subpopulation was 143 ± 125 cm^2^ with no significant difference seen between the two groups (142 ± 119 cm^2^ in those who received the TAP block vs. 142 ± 146 cm^2^ in those with no TAP block, P = 0.76).

**Table 2:** Demographics - postoperative opioid use ʄKruskal-Wallis test; ɸ Pearson's chi-squared; ǂAnalysis of variance test TAP, transversus abdominis plane

Characteristic	N	TAP Block, N = 214	No TAP Block, N = 67	Combined, N = 281	Test Statistic
Age (median [Q1-Q3])	281	57 (47–66)	56 (46–68)	57 (47–66)	P = 0.76^ʄ^
Advanced age (>70 years)	281	16% (34)	19% (13)	17% (47)	P = 0.62^ɸ^
Gender	281				P = 0.05^ɸ^
Female		60% (128)	73% (49)	63% (177)	
Male		40% (86)	27% (18)	37% (104)	
Number of previous abdominal surgeries (mean ± SD)	281	2.52 ± 2.11	3.10 ± 2.13	2.66 ± 2.13	P = 0.03^ɸ^
Hernia diameter in cm (mean ± SD)	281	12.5 ± 5.0	12.5 ± 5.2	12.5 ± 5.0	P = 0.42^ǂ^
Hernia area in cm (mean ± SD)	281	142 ± 119	142 ± 146	143 ± 125	P = 0.76^ǂ^
BMI (mean ± SD)	281	34 ± 7	34 ± 8	34 ± 7	P = 0.50ǂ

Postoperative LOS

The average LOS following AWR was 1.54 ± 1.50 days. Patients who received the multimodal TAP block had an average hospital LOS, which was significantly shorter than patients who did not have a TAP block (1.09 ± 1.22 vs. 2.53 ± 1.57 days, P < 0.001). Of the 17 patients with a prolonged LOS (>4 days postoperatively), five received a TAP block and 12 did not receive a TAP block (2.1% vs 11% respectively, P < 0.001) (Table 3).

**Table 3:** Univariate analysis - hospital LOS *P < 0.05 TAP, transversus abdominis plane; LOS, length of stay

	TAP Block, N = 235	No TAP Block, N = 109	Combined, N = 344	P-value
LOS (mean ± SD)	1.09 ± 1.22	2.53 ± 1.57	1.54 ± 1.50	<0.001^*^
Prolonged LOS (>4 days)	2.1% (5)	11% (12)	4.9% (17)	<0.001^*^

Postoperative opioid requirements

Table 4 provides a summary of analyses for the type of opioid required to control postoperative pain. Following AWR, 11% of patients required a hydromorphone PCA pump, 41% required IV opioids, and 41% required oral opioids. In addition, 9.3% of patients required a combination of hydromorphone PCA pump plus oral opioids and 17% required a combination of IV opioids plus oral opioids. Comparing patients who received a TAP block to those who did not receive a TAP block significantly, fewer patients required hydromorphone PCA (3.3% vs 36%, P < 0.001), oral opioids (29% vs 78%, P < 0.001), and a combination of hydromorphone PCA pump and oral opioids (1.9% vs 33%, P < 0.001). Significantly more patients with the TAP block required IV opioids (50% vs 10%, P < 0.001) and a combination of IV opioids and oral opioids (21% vs 6%, P = 0.005) when compared to patients who did not receive the TAP block.

**Table 4:** Univariate analysis - postoperative opioid use *P < 0.05 TAP, transversus abdominis plane; PCA, patient-controlled analgesia; IV, intravenous; PRN, pro re nata (as needed)

	TAP Block, N = 214	No TAP Block, N = 67	Combined, N = 281	P-value
Hydromorphone PCA pump	3.3% (7)	36% (24)	11% (31)	<0.001^*^
IV opioids PRN	50% (108)	10% (7)	41% (115)	<0.001^*^
Oral opioids PRN	29% (62)	78% (52)	41% (114)	<0.001^*^
PCA pump + oral opioids	1.9% (4)	33% (22)	9.3% (26)	<0.001^*^
IV opioids + oral opioids	21% (45)	6.0% (4)	17% (49)	0.005^*^

On further analysis of opioid requirements, the mean IV morphine dosage required by the cohort was 5.19 ± 2.99 mg. Patients who received the TAP block required an average of 4.86 ± 2.62 mg and patients who did not receive the TAP block required an average of 10.29 ± 3.90 mg (P < 0.001). The mean dosage of oral oxycodone required was 30 ± 30 mg, with a statistically significant difference seen between the TAP block group and the non-TAP block group (24 ± 22 mg vs 37 ± 36 mg, P = 0.006) (Table 5).

**Table 5:** Postoperative opioid dosage requirements *P < 0.05 TAP, transversus abdominis plane; IV, intravenous

Univariate Analysis - IV Morphine	TAP Block, N = 108	No TAP Block, N = 7	Combined, N = 115	P-value
IV morphine mg (mean ± SD)	4.86 ± 2.62	10.29 ± 3.90	5.19 ± 2.99	<0.001^*^
Univariate Analysis - Oral Oxycodone	TAP Block, N = 62	No TAP Block, N = 52	Combined, N = 114	P-value
Oral oxycodone mg (mean ± SD)	24 ± 22	37 ± 36	30 ± 30	0.006^*^

## Discussion

The use of local analgesic TAP blocks in gastrointestinal surgery, including hernia repair, has been previously demonstrated to decrease postoperative morphine requirements; however, even with the administration of TAP blocks, patients undergoing hernia repair require significant postoperative pain management and lengthy hospital stays following surgery [1,18,20]. TAP blocks traditionally consist of a single local anesthetic; however, orthopedic operations have demonstrated increased efficacy in the direct blockade of the neural afferent supply using multimodal nerve blocks [10]. In this study, the efficacy of a relatively novel multimodal TAP block used during AWR was assessed. It was observed that the multimodal TAP block containing ropivacaine, epinephrine, and ketorolac proved effective in decreasing opioid need postoperatively as well as decreasing the hospital LOS following elective hernia repair for large ventral hernias.

Previous meta-analysis data have demonstrated that TAP blocks significantly reduce cumulative opioid requirements when compared to patients who did not receive a TAP block [1,21]. Gao et al. [1] published a meta-analysis including five studies, three open hernia repairs and two laparoscopic hernia repairs, all of which reported a significant decrease in opioid usage postoperatively when TAP blocks were used. Fields et al. [22] demonstrated a 40% decrease in morphine requirements postoperatively following laparoscopic hernia repair in patients who received a TAP block of 0.25% bupivacaine compared to those who did not. While the TAP block proved effective in reducing opioid requirements overall, patients on average required 25.64 mg of morphine at 24 hours postoperatively [22].

Epinephrine, when bound to alpha receptors, causes vasoconstriction inducing a quicker onset of action of the local anesthetic and reducing the likelihood of local anesthetic toxicity. In addition, vasoconstriction extends the duration of the analgesia by delaying the systemic uptake of the anesthetic [6,13]. Epinephrine, while beneficial, must have dosages used in limitation due to the negative effect vasoconstriction can have on postoperative wound healing [6]. NSAIDs have anti-inflammatory and antipyretic properties and have been proven to have similar analgesic efficacy to that of typical dosages of morphine and meperidine without the additional side effects [14]. The side effects of postoperative opioids are well understood and include nausea, vomiting, ileus, and urine retention, among others [23]. These side effects delay the discharge of patients and induce preventable complications, which are costly and increase the risk of postoperative complications. A combination of non-narcotic medications, such as NSAIDs, specifically in perioperative pain management has been proven to reduce pain intensity, nausea, duration of ileus, and the average LOS after abdominal surgery [12,15-17].

Hospital LOS has not been consistently proven to decrease in patients receiving a traditional unimodal TAP block during gastrointestinal surgery. Hamid et al. [21] published a meta-analysis that included three studies reporting hospital LOS following bariatric surgery and found no significant difference in hospital LOS in patients who received a traditional TAP block compared to those who did not. Fields et al. [22] demonstrated no significant difference in hospital LOS in patients receiving a TAP block versus placebo during laparoscopic hernia repair; however, Warren et al. [20] demonstrated a significantly shorter hospital LOS (2.4 vs 4.5 days; P < 0.001) in patients undergoing ventral hernia repair and receiving a TAP block versus epidural analgesia. The study by Fields et al. [22] included only laparoscopic hernia repair while Warren et al. [20] only included open hernia repair, demonstrating that current effectiveness of TAP block is dependent on surgical technique.

These previous studies suggest that while a traditional TAP block containing only a local analgesic may be effective in decreasing postoperative pain and opioid requirements, patients are still requiring a considerable number of opioids and are continuing to experience inconsistent postoperative hospital stays.

This retrospective study demonstrated significantly decreased hydromorphone PCA pump usage and oral opioid usage following elective robotic AWR for large ventral hernias in patients who received a multimodal TAP block. While significantly more patients required IV opioids (morphine) postoperatively, postoperative morphine dosages were significantly decreased in patients who received a TAP block. The increase in IV opioids in patients with the TAP block may also be explained due to the sizable proportion of patients without the TAP block requiring a hydromorphone PCA pump, thus not requiring IV opioids. This study also exhibited a significantly shorter hospital LOS in patients who received the multimodal TAP block compared to patients who did not.

While the results of this study are promising, there are limitations and a need for future studies. This study contained patients only undergoing one type of gastrointestinal surgery. While this may have decreased potential confounding variables without future studies, the efficacy of this multimodal TAP block on other gastrointestinal surgeries is unknown. In addition, future studies comparing a traditional TAP block of a single analgesic to the multimodal TAP block will be beneficial now that initial efficacy has been proven. The significantly shorter hospital LOS seen in patients with the TAP block may be explained due to the decreased opioids used leading to decreased opioid side effects; however, future studies will be required to confirm.

## Conclusions

In conclusion, this novel multimodal TAP block of ropivacaine, epinephrine, and ketorolac was found to decrease hospital LOS and postoperative opioid requirements in patients undergoing elective robotic AWR for large ventral hernias. These findings are in line with previous studies that have shown general effectiveness in reducing opioid requirements but show further decreases in dosage as well as differences in hospital LOS, which have not been seen in the past. Future studies using various populations for different surgical procedures as well as studies comparing the novel TAP block to the traditional TAP block will be required to provide feasibility and efficacy.
